# Protection of HEVC Video Delivery in Vehicular Networks with RaptorQ Codes

**DOI:** 10.1155/2014/619379

**Published:** 2014-07-17

**Authors:** Pablo Piñol, Miguel Martínez-Rach, Otoniel López, Manuel Pérez Malumbres

**Affiliations:** Physics and Computer Architecture Department, Miguel Hernández University, Avenida de la Universidad, s/n, 03202 Elche, Spain

## Abstract

With future vehicles equipped with processing capability, storage, and communications, vehicular networks will become a reality. A vast number of applications will arise that will make use of this connectivity. Some of them will be based on video streaming. In this paper we focus on HEVC video coding standard streaming in vehicular networks and how it deals with packet losses with the aid of RaptorQ, a Forward Error Correction scheme. As vehicular networks are packet loss prone networks, protection mechanisms are necessary if we want to guarantee a minimum level of quality of experience to the final user. We have run simulations to evaluate which configurations fit better in this type of scenarios.

## 1. Introduction

Our lives have experimented a radical change since cell phones are no longer just telephones and have become smartphones, with good processing capabilities, large store capacity, and above all, great connectivity. This connectivity allows us to have all kinds of information available at every moment. And we are not only information consumers but active producers as well. At the day when vehicular networks will be integrated by a high percentage of our vehicles and infrastructures in our cities and roads, a vast number of applications of all types (some of them nowadays unthinkable) will arise. Many of them will be oriented to safety and others to entertainment, economizing, and so forth. Within vehicular networks applications, video streaming can be very useful. But, on the one hand, vehicular networks are inhospitable environments where packet losses appear, and on the other hand, video transmission is heavily resource demanding. The combination of these two characteristics makes video streaming over vehicular networks a hard to manage task.

The aim of this work is to evaluate the protection of video encoded with the emerging standard High Efficiency Video Coding (HEVC) by using RaptorQ codes and how they both behave in vehicular scenarios. For this task we will vary a series of parameters in both HEVC and RaptorQ and will present the performance of those configurations.

There are lots of works that evaluate HEVC efficiency (like [1, 2]) although most of them do not take into consideration lossy environments. Some works evaluate HEVC performance under packet loss conditions as the authors do in [3]. In that work the authors have developed a complete framework for testing HEVC under different packet loss rates, bandwidth restrictions, and network delay. As HEVC decoder is not robust against packet losses (it crashes if packets are missing) their framework decodes the complete bitstream and then overrides the areas corresponding to the missing packets. Several works propose mechanisms for protecting video streaming over vehicular networks although quality evaluation refers to percentage of lost packets [4]. In [5] the authors do a thorough research of protection of content delivery in vehicular environments searching for the best packet size in order to maximize throughput. They use different FEC techniques to protect data and offer results in terms of packet arrival ratio and file transfer time.

Our work differs in some aspects from the cited research works. The main difference is that our work combines several features of the cited works into one research. And also it includes features that are not included in previous works. In our work we have used real maps to design our vehicular scenarios. We have not used synthetic losses but the real losses obtained by simulations of vehicles moving through the scenario. We have used HEVC reference software with some modifications in order to make it robust against packet losses (avoiding crashes when packets are lost). So we have decoded the bitstream with missing packets directly with the reference software. We have used RaptorQ codes in order to protect the video stream and we have tuned them to test which configurations get better results. And not only statistics about percentage of recovered packets are provided, but also the quality of the real reconstructed video sequence is presented (in terms of PSNR versus the original sequence).

The rest of the paper is structured as follows. In Section 2 the three main components of the scenarios are presented: vehicular networks, HEVC, and RaptorQ codes. In Section 3 the framework where the tests have been done is explained.  Section 4 explains the results obtained for the different configurations that we have tested. At last, in Section 5, several conclusions are drawn.

## 2. Components

In this section we describe the three integral parts of the scenarios of our research: vehicular networks, HEVC, and RaptorQ codes.

### 2.1. Vehicular Networks

In future, vehicles will be equipped with lots of sensors (nowadays they already are) which will be able to collect internal and external measurements. They will also be provided with a certain computing capability which will allow them to process information, and they will also carry communications equipment. These three elements will make Intelligent Transport Systems (ITS) possible, where vehicles will have the ability to communicate with each other and with infrastructure in an intelligent way. This capacity of vehicles to communicate with each other and with a fixed network will bring both vehicle-to-vehicle (V2V) and vehicle-to-infrastructure (V2I) networks. These networks will be used by applications regarding areas like people safety, fuel consumption savings, reduction of CO_2_ emissions, infotainment, and so forth. Video streaming will be used by diverse types of applications like digital entertainment, Video On Demand (VOD), tourist information, contextual advertising, traffic flow density, and other regarding safety issues like emergency video call and so forth. Vehicular networks will have many challenges due to their nature. The combination of wireless communications with the relative high speed of nodes, variability of routes, and obstacles disturbing wireless signal (buildings, other vehicles, etc.) will make them packet loss prone networks. In packet loss prone networks, video streaming applications, which produce big deals of data and have high bandwidth requirements, may decrease the network performance and may have to deal with high rates of packet loss. These big deals of data need to be efficiently compressed to diminish the bandwidth needed for streaming. So we will need the assistance of proficient video codecs.

### 2.2. HEVC

On January 2013, High Efficiency Video Coding [6] was agreed upon as the new video coding standard. By 2010, ITU-T Video Coding Experts Group (VCEG) and ISO/IEC Moving Pictures Experts Group (MPEG) joined their research efforts and constituted the Joint Collaborative Team on Video Coding (JCT-VC) in order to develop a new video coding standard that would improve the previous one, H.264/AVC (Advanced Video Coding) [7], and could keep pace with the growing resolutions and frame rates of new video contents. The new standard follows the same hybrid compression scheme as its predecessor but includes a good number of refinements and new features that nearly doubles its coding efficiency. Here we will explain one of the features of HEVC that we will use in our tests: slices. For a deeper insight into the standard, some of the members of JCT-VC provide a complete overview in [8].

Slices are components of an encoded video stream that were introduced in the previous video coding standard, H.264/AVC. They are coded fragments of a frame that can be independently decoded. This makes them especially useful in packet loss prone scenarios. If we encode each frame of a video sequence using only one slice and that slice is bigger than the network MTU, then we will have to divide the slice in several fragments which will travel in several network packets. If one of these packets gets lost, then the rest of the fragments of the slice will become completely useless, because a slice cannot be decoded if it is not complete. In this way the loss of a single packet implies the effective loss of the whole frame. But if we divide each frame into several slices and each slice is smaller than the network MTU, then we will be able to send each slice in one packet. The loss of a single packet would not imply the loss of the whole frame but only the loss of a slice because the rest of the packets could be independently decoded.

But there is a drawback in dividing a frame into several slices. As slices are independently decodable, prediction is not allowed farther away of the slice limits. For instance, spatial prediction using areas of other slices is not allowed. The same happens to motion vectors prediction that must remain inside slice limits. This causes a decrease in coding efficiency. For every slice we will have a slice header which will also introduce some overhead. And if we divide a frame into too many slices, then the size of each slice may be much smaller in comparison with other headers used for streaming (e.g., RTP) and this would introduce a considerable overhead. In this work we will study how HEVC behaves in video streaming over vehicular networks for different number of slices per frame with the aid of RaptorQ codes to deal with packet loss.

### 2.3. RaptorQ Codes

Raptor codes, invented by Shokrollahi [9, 10], are a type of Fountain codes and are based in Luby Transform (LT) codes [11]. Raptor codes are a Forward Error Correction (FEC) technology which implements application layer protection against network packet losses. RaptorQ codes are a new family of codes that provide superior flexibility, support for larger source block sizes, and better coding efficiency than Raptor codes. They have several features that make them an interesting technology. One of them is that they can encode (protect) and decode (restore) data with linear time. They can also add variable levels of protection to better suit the protection to the network characteristics (e.g., packet loss ratio, maximum bandwidth, etc.). They have very good recovery properties, because they can completely recover the original data if they receive approximately the same amount of data than the original one, regardless of whether the received packets are original packets or repair packets. RaptorQ codes are very efficient and have small memory and processing requirements, so they can be used in a wide variety of devices (from smartphones to big servers). Raptor and RaptorQ codes have been standardized by IETF [12, 13] and are used in 3GPP Multimedia Broadcast Multicast Services (MBMS) for file delivery and streaming.

This is how RaptorQ operates. The RaptorQ encoder receives a data stream (source packets) during a specified protection period. These packets are put together in memory to form a source block. A 4-byte FEC trailer is added to each received packet. This trailer identifies the packet and the protection period to which it belongs. These FEC-protected packets are sent through the network. When the protection period finishes, the source block in memory is FEC-encoded into repair symbols which are placed into repair packets and sent through the network. At the receiver side, the RaptorQ decoder receives protected source packets and repair packets. Some of these packets may get lost or corrupted and the RaptorQ decoder will try to recover lost packets out of the group of source and repair packets correctly received.

Latency (and, in some cases, memory consumption) is the drawback of RaptorQ protection scheme. As the protection window increases, the delay grows. Live events or real-time applications like video conference will have to use short periods for the protection window to keep latency into reasonable limits. Other types of streaming applications like IPTV may tolerate wider protection windows (while keeping latency inside a reasonably interaction response time). And some other applications like Video On Demand can be much more flexible in enlarging the protection period which will provide more bandwidth efficiency.

## 3. Framework

In our tests, several tools and simulators have been used. For the construction of the vehicular scenario we have used the OpenStreetMap project [14]. The OpenStreetMap project is a public domain geographic data base, built upon contributions of volunteers of all around the world. It is a project like Wikipedia but with geographic data, where you can find varied information like streets (including the number of lanes and their direction), parks, squares, schools, bus stops, singular buildings, drugstores, rivers, and so forth. From this page we have downloaded a real map of the city of Kiev. This map (XML data) has to be converted in order to be handled by SUMO (simulation of urban mobility) [15]. SUMO is a traffic simulator which is well known by the scientific community. It is able to run traffic simulations including vehicles, traffic lights, crossroads, priorities, and so forth and allows defining characteristics like the size, acceleration, deceleration and maximum speed of vehicles, and gathering different statistics like fuel consumption and CO2 emissions. One of the tools which is included in SUMO is TraCI (Traffic Control Interface). By using this interface, a bidirectional communication is possible between SUMO and other applications. In our tests we will connect SUMO (which will run the simulation of vehicles mobility) with OMNeT++ [16] where the vehicular network will be tested.

OMNeT++ is not a simulator itself but a framework for the development of simulators. It is a public domain software and a lot of projects (which implement the actual simulators) are available for it. Two of these projects have been put together to provide a vehicular network simulator, MiXiM [17] and Veins [18]. MiXiM is a project which implements wireless communications both fixed and mobile. Veins adds some protocols which have been standardized for vehicle-to-vehicle (V2V) and vehicle-to-infrastructure (V2I) wireless communications, like IEEE 802.11p [19] and the IEEE 1609 family of standards [20].

Inside this vehicular network simulator we have developed an application for driving the experiments. It basically allows the injection of a video stream and the insertion of background traffic to produce congestion in the network to simulate adverse conditions. It also gathers some statistics for subsequent analysis.

For the encoding and decoding of HEVC video we have used the HEVC reference software [21]. That software has been modified by us in order to generate RTP packets inside the bitstream. In addition we have modified the HEVC decoder to make it resilient to packet losses. We have used two different encoding modes, specified by the JCT-VC. On the one hand we have used All Intra (AI) mode, in which every frame of the video sequence is encoded as an I frame. An I frame is encoded wihtout using other frames as a reference, and therefore, it is independent of the rest of the frames. This is called intramode. An I frame exploits the spatial redundancy that exists inside the frame. On the other hand, we have used Low-Delay P (LP) mode. In this mode the first frame is encoded as an I frame and the rest of the frames are P (predictive) frames. P frames use other frames previously encoded (and decoded) as a reference to exploit temporal redundancy. They use motion estimation/compensation. This is called inter mode. Inter mode is more efficient than intramode; thus, better compression rates are obtained by LP encoding mode than by AI encoding mode. In LP mode, P frames depend on other frames used as a reference. This makes this mode more vulnerable to packet losses. In AI mode the loss of a slice will only affect one frame, but in LP mode, the loss of a slice will affect the frame which it belongs to and also the frames that use this frame as a reference. The frames that use these affected frames will also be damaged and so on. To stop this drift and try to alleviate packet losses (those that could not be recovered with RaptorQ codes) we have used the intra-refresh mechanism in LP encoded streams. This mechanism consists basically in inserting an I frame every 32 frames. If all the slices that belong to this frame arrive to their destination (or equivalently, RaptorQ codes are able to recover them) the sequence is refreshed and the propagation of errors ends.

For the protection of the bitstream with RaptorQ codes we have used the Qualcomm (R) RaptorQ (TM) Evaluation Kit [22]. RaptorQ software has different options to better tune it and suit it to fit your needs or preferences. For instance, you can specify the amount of protection to add to the bitstream (i.e., 10%, 20%, etc.). The bigger the amount of protection you add, the higher the probability of successful recovery of lost packets will be, but also the higher the bandwidth required for the transmission will become. You can also adjust the length of the temporal window which will divide the bitstream in fractions in order to generate FEC packets. You can also set the symbol size and the repair packet size. An adequate symbol size can make the encoding and decoding of FEC data more computationally efficient and also reduce the amount of memory required for these computations. The final aim of this work is to evaluate how HEVC and RaptorQ codes behave and how they can collaborate to protect video transmissions in vehicular networks and determine which are the most suitable configurations of both in each situation. This is a necessary step to propose adaptive mechanisms that can optimize resources and provide error resilience techniques that assure a good quality of experience in video streaming via vehicular networks.

For the performance of the tests we have followed these steps. Once the scenario has been implemented, we have encoded a raw video sequence at different number of slices per frame producing several HEVC bitstreams (in RTP format). Each one of these bitstreams has been FEC encoded with several configurations using RaptorQ. Protected sequences have been used to run simulations. Each simulation produces a file with several statistics (including the packet loss ratio) and a file with the received packets. This file is FEC decoded by means of RaptorQ in order to try to recover lost packets and to produce an HEVC file (in RTP format). This RTP file is decoded by HEVC decoder and finally the reconstructed raw video sequence is compared to the original one in order to calculate PSNR and evaluate final video quality.

In next section we will give some extra details about tests and we will analyze the most relevant results obtained.

## 4. Experiments and Results

**Figure 1:**
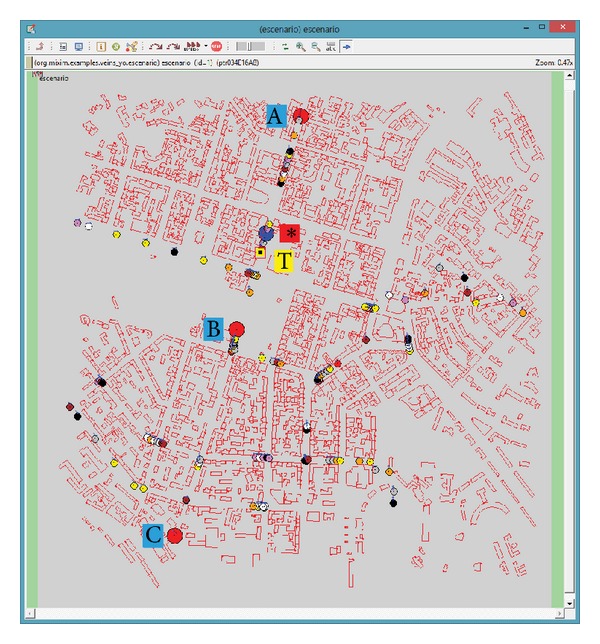
Vehicular network scenario in OMNeT++ (red circles A, B, C = RSUs//blue circle ∗ = video client//yellow square T = background traffic source//small circles = other vehicles//red rectangles = buildings).

The study case is based on the scenario shown in Figure 1. It is an area of 2000 m × 2000 m of the city of Kiev. In it we can find a long avenue that crosses that area from north to south. Along the avenue, 3 road side units (RSUs) have been positioned, named A, B, and C in the figure. These RSUs will transmit the video sequence simultaneously (in a synchronized way). The coverage radius of the wireless devices is 500 m. RSUs A and B have a small area where their signals overlap. And RSUs B and C have a small shaded area where neither of the two can reach. Therefore we have three different types of areas regarding transmission: areas where a vehicle can receive the data from only one RSU, one area where the vehicle receives the signal from two RSUs (A and B), and one area where signal is momentarily lost (between B and C coverage areas). A total of 50 vehicles have been inserted into the scenario, driving in different routes. Those vehicles send a beacon every second through the control channel (following IEEE 1609.4 multichannel operations). We also have a vehicle driving near the video client that can act as a background traffic source (labeled as T in Figure 1), sending packets through the wireless network at different packets per second (pps) rates. The video client (marked in the figure as ∗) will experience isolated packet losses (mainly due to background traffic) and bursty packet losses (around the limits of RSUs coverage). RSUs send periodically through the control channel advertisements of the video service that they offer, indicating the service channel used for the video stream. The video client receives that invitation and commutes to the specified service channel in order to receive the video stream.

### 4.1. HEVC Evaluation

Now we present the evaluation of the video sequence behavior when it is encoded at a different number of slices per frame in both encoding modes used (AI and LP).

**Figure 2:**
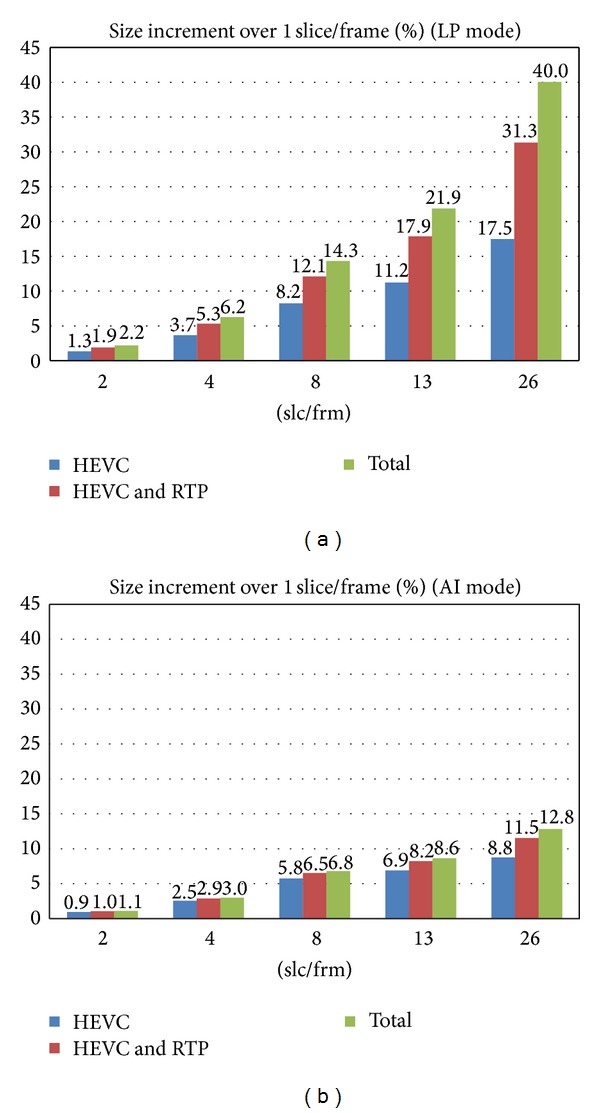
Percentage of bitrate increase (without FEC protection) for different number of slices per frame. (a) LP mode. (b) AI mode. (HEVC raw bitstream//HEVC + RTP header//HEVC + RTP header + fragmentation header.)

The sequence chosen for the tests is RaceHorses, one of the test sequences used by JCT-VC for HEVC evaluation in common test conditions [23]. It has a resolution of 832x480 pixels and a frame rate of 30 frames per second. We have encoded it at 1, 2, 4, 8, 13, and 26 slices per frame (slc/frm). For the encoding process we have used a value of 37 for the quantization parameter (QP). For that value we obtain a mean PSNR value of 32.12 dB for AI mode (1 slc/frm) and a value of 30.19 dB for LP mode (1 slc/frm). Video quality is higher in AI mode but bitrate in LP mode is much lower. As predictions cannot cross slice boundaries, when we split each frame into several slices we are reducing coding efficiency because we cannot use information of nearby areas if they do not belong to the slice. An example of this penalization is that slices cannot use intraprediction between slices (in AI mode) and slices cannot use predictions for motion vectors (in LP mode).  Figure 2 shows the percentage of increment of the encoded sequence size at different number of slices per frame compared to 1 slc/frm. It shows the percentage of increment of HEVC raw bitstream and also the percentage of increment after adding the RTP headers to each slice. If the size of one slice (including its RTP header) is greater than the network MTU, then it will be divided into some fragments. If one of the fragments of a slice gets lost, then the whole slice will be discarded because it will be undecodable. So the rest of the fragments of the slice are automatically discarded. We identify every fragment of a slice with a header in order to know if a slice has received all its fragments. In Figure 2, data labeled as “TOTAL” shows the bitrate increment with respect to 1 slc/frm when both RTP and fragmentation headers are included. As slices generated by LP mode are much smaller than slices generated by AI mode (because of LP mode coding efficiency), the overhead introduced by RTP and fragmentation headers is much greater for LP mode, and consequently the same happens to the total percentage of increment in the bitstream. For example, encoding at 13 slc/frm increases the bitstream around a 20%, and encoding at 26 slc/frm increases it around a 40% for LP mode. But for the same number of slices per frame, in AI mode the increments are around 8.6% and 12.8%, respectively.

**Figure 3:**
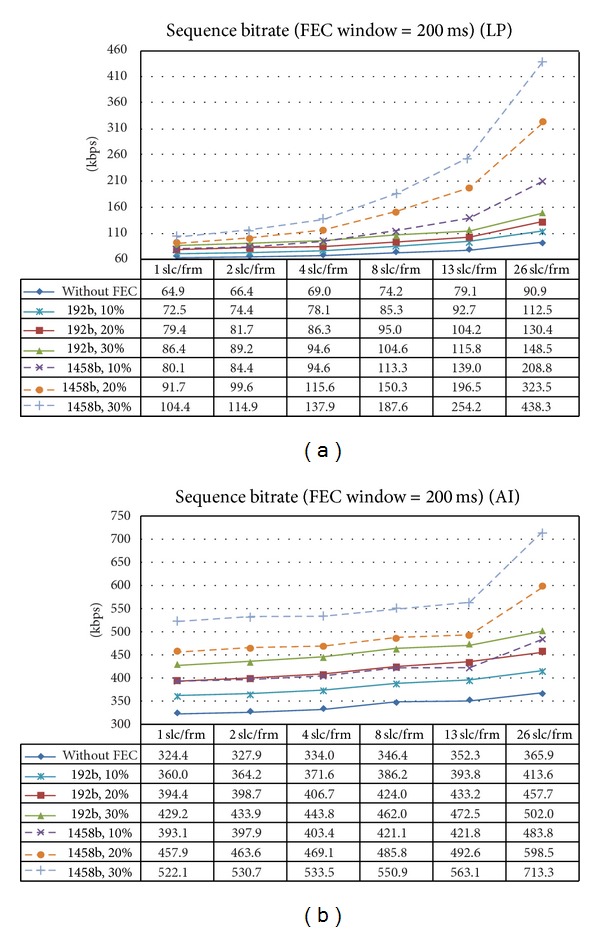
Bitrate (kbps) without FEC protection and with FEC protection for different symbol sizes, different protection windows, and different number of slices per frame (including RTP and fragmentation headers). (a) LP mode. (b) AI mode.

In Figure 3, if you look at the curve labeled “without FEC,” you can see the bitrate value (not the percentage of increment) of the sequence for LP and AI modes. In LP mode the bitrates range from 64.9 kbps (1 slc/frm) to 90.9 kbps (26 slc/frm). In AI mode the bitrates range from 324.4 kbps (1 slc/frm) to 365.9 kbps (26 slc/frm). As we stated before in this section, the bitrate for mode LP is much lower than for AI mode; specifically, at 1 slice per frame the bitrate of LP mode is 5 times lower than AI's one.

A factor that can be of great relevance when protecting data is the proportion of network packets (fragments) with respect to RTP packets (each RTP packet includes one slice). As stated before, if we have many fragments for one RTP packet, then the probability of losing this RTP packet increases, because the real loss of only one of the fragments will render the RTP packet useless. And this will result in the effective loss of the rest of the fragments of that RTP packet. But if we have one fragment per RTP packet, then the loss of one fragment will not affect the rest of packets. When the proportion tends to 1 every lost packet which can be recovered by RaptorQ is contributing to improve the final quality of the reconstructed video. When the proportion moves far away from 1 the recovery of packets by RaptorQ does not always directly turn into an improvement on video quality.

The mean proportion of fragments per RTP packet is shown in Table 1. Data show that when using AI mode and few slices per frame the proportion is far from 1, and this indicates that packet recovery will not be as productive as for LP mode. Data of Table 1 will be different if we encode other video sequences with larger (or smaller) resolutions or with different values for QP parameter or with different values for intra-refresh period. The conclusion is that before protecting video streams with RaptorQ codes, we should take into consideration this proportion in order to better decide the suitable number of slices per frame that will take a greater advantage of FEC protection.

### 4.2. RaptorQ Codes Setup

For the evaluation of RaptorQ and determining which configurations are most suitable, we have protected each of the generated bitstreams with different setups for RaptorQ. 6 different sizes for the temporal window have been used (133, 200, 250, 333, 500, and 1000 milliseconds), 3 different levels of protection have been assigned (10%, 20% and 30%), and 2 different combinations of symbol size and repair packet size have been used (a small symbol size of 192 bytes together with a repair packet size of 7 and a big symbol size of 1458 bytes together with a repair packet size of 1).

First of all we have analyzed the overhead generated by the protection added with RaptorQ codes. In addition to the global bitrate increment we have measured the increment in the packet rate (which is caused by the addition of repair packets). In previous works we found out that in vehicular networks packet losses are more influenced by packet rate than by packet size, so if we do not keep the extra packets per second low, then the solution to our problem (adding extra repair packets) can become the problem of the network.

Table 2 shows the number of packets per second in the transmission of the LP encoded sequence without any FEC protection and also with FEC protection with a redundancy of 30% for different symbol sizes and different temporal windows. When we use a big symbol size, more packets per second are generated than when we use a small symbol size. This gets worse as the number of slices increases. It can also be seen that varying the temporal window does not change significantly the packet rate.

Table 3 shows the packet rate without FEC protection and by using FEC protection with a symbol size of 192 bytes and a temporal window of 200 ms. If we compare the number of packets per second for LP and AI modes without using FEC we can see the correlation with Table 1. When we encode the video sequence at 26 slc/frm, we have a proportion of 1.00 fragment per RTP packet for both LP and AI modes. This means that every single RTP packet is smaller than the MTU and the packet rate can be directly calculated by multiplying the frame rate (30 fps) by the number of slices per frame (26 slc/frm); this is 780.0 pps. At the opposite side of Table 3, if we look at AI mode without using FEC encoded at 1 slc/frm, we can see that the packet rate (237.0 pps) is very far from the multiplication of the frame rate (30 fps) by the number of slices per frame (1 slc/frm). At 1 slc/frm AI mode and LP mode produce very different packet rates. Observing FEC-protected data it can be seen that when fewer slices per frame are used the proportion of extra packets per second is greater.

Figure 3 shows the bitrate (kbps) of the encoded sequences in modes LP and AI without FEC protection and with FEC protection for three different levels of redundancy and two symbol sizes. Selecting 192 bytes as the symbol size leads to much lower bitrates. This is more emphasized for LP coding mode.

### 4.3. OMNeT++ Tests

In this section we will present the tests performed in the simulations. For the experiments we have used the framework previously depicted, using SUMO, OMNeT++, MiXiM, and Veins.

In simulations we connect SUMO and OMNeT++ via TraCI. SUMO tells OMNeT++ the position of every vehicle at every instant and OMNeT++ (using MiXiM and Veins) runs the simulation of the vehicular network. Video sequences which have been previously protected with RaptorQ codes are transmitted from the 3 RSUs to the video receiver. At the end of the simulation a file is generated with some statistics, including the percentage of packets lost. Another file is also generated including the packets received by the vehicle. This file is processed using RaptorQ decoder in order to generate a file with RTP packets, trying to recover the lost packets. The output of this process is passed to the HEVC decoder which will try to restore the video sequence. After this process we compute the PSNR value for the reconstructed sequence.

We have run simulations for the following combinations: both modes of encoding (AI and LP), different number of slices per frame (1, 4, 8, and 13 slc/frm), and three protection levels (10%, 20%, 30%). For all these combinations we have run tests injecting background traffic at four different rates (0, 30, 240, and 390 pps).

In areas of full coverage, packet losses are due to background traffic. Here we encounter isolated losses. On the contrary, in areas near the limits of the RSUs coverage, packet losses are bursty because the signal is completely lost for some period. For isolated losses RaptorQ codes do a good job in recovering lost packets. Tables 4 and 5 show the total percentage of network packet loss, the percentage of RTP packet loss after recovery, and the difference in PSNR of the reconstructed video for LP and AI modes, respectively (for areas of good coverage). As it can be seen, RaptorQ can recover a high percentage of network packets and the result is that only a small percentage of RTP packets are lost. This is not true for AI mode and few slices per frame (1 slc/frm and 2 slc/frm), but this is the expected behavior taking into account that, as we stated before, in these configurations the proportion of fragments per RTP packet is high. We can observe from those tables that AI mode is inherently more error resistant than LP mode. This is also the expected behavior because in LP mode reference pictures with incorrect data propagate errors and in AI mode errors do not propagate. Some techniques like unequal error protection methods could be useful to introduce different levels of protection regarding the importance of the video packets (I or P frames) in the final quality of the reconstructed sequence. Regarding areas near the limits of RSUs coverage, we can state that RaptorQ is not able to deal with bursty losses because within the protection period very few packets are received and there is not enough data to carry out a recovery. This problem could be addressed with the introduction of techniques like interleaving, where, with the trade-off of the introduction of some delay, bursty losses can be easily converted to isolated losses (where RaptorQ can get good percentages of recovery). We can see that a symbol size of 1458 bytes provides better recovery results but if we take into consideration data from Figure 3, then the overhead introduced is not bearable. At last, from the very specific conditions of our tests we can state that the optimum number of slices per frame is 8, which produces the best trade-off between the introduced overhead (both packets per second and total bitrate) and the percentage of recovery and the final video quality.

## 5. Conclusions

In this work we have analyzed the protection of video delivery in vehicular networks. The video encoder selected for the tests is the new emerging standard HEVC. To protect the video stream we have used RaptorQ codes. We have first analyzed the behavior and performance of HEVC for two coding modes (AI and LP) and for different number of slices per frame. Then we have protected the encoded sequences by means of RaptorQ with several configurations and observed the effects of this selection. We have seen that varying HEVC and RaptorQ parameters leads to very different situations, regarding total bitrate and packet rate. At last we have run simulations in a vehicular environment to measure the ability of RaptorQ in protecting video packets in this type of scenarios. The reduction in the number of lost packets because of RaptorQ codes recovery properties has been presented as well as the quality of the decoded video reconstructions. As a general conclusion we can state that there are a lot of parameters that can be fine-tuned to adapt the video protection to the requirements of the specific network conditions (bandwidth, packet loss ratio, isolated/bursty losses, etc.) and to the requirements of the specific application (encoding mode, level of quality, resolution of video sequence, etc.). So there is not a general formula which will fit into all situations to provide the best level of protection. On the contrary, a previous evaluation of the real conditions and user preferences is mandatory.

## Figures and Tables

**Table 1:** Mean proportion of fragments (network packets) for every RTP packet (slice).

Fragments/RTP	1 sl	2 sl	4 sl	8 sl	13 sl	26 sl
LP mode	1.87	1.37	1.21	1.02	1.00	1.00
AI mode	7.90	4.29	2.38	1.51	1.07	1.00

**Table 2:** Packet rate (packets per second) for LP mode without FEC protection and with FEC protection at 30% of redundancy for different symbol sizes, different protection windows, and different number of slices per frame.

Packets/sec.	1 sl	2 sl	4 sl	8 sl	13 sl	26 sl
Without FEC	56.1	82.0	134.6	244.9	391.3	780.0

192 b, 133 ms	75.4	102.7	156.2	270.0	420.7	824.6
192 b, 200 ms	73.8	100.5	155.0	268.7	419.6	822.7
192 b, 250 ms	73.1	100.1	154.6	268.4	419.0	821.5
192 b, 333 ms	72.9	99.8	154.2	267.7	418.4	820.7
192 b, 500 ms	72.1	99.2	153.3	266.5	417.0	821.5
192 b, 1000 ms	71.2	97.9	152.0	265.2	415.5	820.9

1458 b, 133 ms	83.2	116.3	183.0	320.4	509.1	1016.7
1458 b, 200 ms	82.9	114.9	181.3	321.7	509.8	1015.1
1458 b, 250 ms	81.8	114.5	180.8	321.6	509.1	1010.0
1458 b, 333 ms	81.8	114.0	180.7	320.0	507.9	1008.9
1458 b, 500 ms	80.5	113.7	179.1	317.8	504.3	1003.9
1458 b, 1000 ms	79.5	111.6	176.2	313.2	498.1	1013.9

**Table 3:** Packet rate (packets per second) without FEC protection and with FEC protection for a symbol size of 192 bytes and a protection window of 200 ms for different coding modes, different percentages of redundancy, and different number of slices per frame.

Packets/sec.	1 sl	2 sl	4 sl	8 sl	13 sl	26 sl
(LP) w/o FEC	56.1	82.0	134.6	244.9	391.3	780.0

(LP) 10%	63.6	89.9	143.3	254.9	402.0	795.4
(LP) 20%	68.5	95.0	148.9	261.9	410.8	808.9
(LP) 30%	73.8	100.5	155.0	268.7	419.6	822.7

(AI) w/o FEC	237.0	257.3	285.3	362.8	417.6	780.0

(AI) 10%	265.0	285.6	314.8	392.9	449.3	815.0
(AI) 20%	290.2	311.0	340.6	421.1	478.2	847.6
(AI) 30%	316.0	336.8	367.7	449.4	507.4	880.7

**Table 4:** Total percentage of network packet loss, percentage of RTP packet loss after recovery, and difference in PSNR of the reconstructed video, for a background traffic of 390 pps and a level of protection of 30%, for areas with good signal coverage. LP encoding mode.

slc/frm	Measurement	192 b	1458 b
1 sl	TOTAL loss (%)	11.28	10.89
1 sl	RTP loss (%)	1.28	0.18
1 sl	PSNR diff (dB)	0.99	0.18

4 sl	TOTAL loss (%)	13.55	14.36
4 sl	RTP loss (%)	1.84	0.62
4 sl	PSNR diff (dB)	2.85	0.75

8 sl	TOTAL loss (%)	14.59	15.05
8 sl	RTP loss (%)	1.47	0.09
8 sl	PSNR diff (dB)	1.90	0.11

13 sl	TOTAL loss (%)	13.97	13.25
13 sl	RTP loss (%)	0.29	0.00
13 sl	PSNR diff (dB)	0.52	0.00

**Table 5:** Total percentage of network packet loss, percentage of RTP packet loss after recovery and difference in PSNR of the reconstructed video, for a background traffic of 390 pps and a level of protection of 30%, for areas with good signal coverage. AI encoding mode.

slc/frm	Measurement	192 b	1458 b
1 sl	TOTAL loss (%)	19.07	19.10
1 sl	RTP loss (%)	14.81	16.94
1 sl	PSNR diff (dB)	2.20	2.52

4 sl	TOTAL loss (%)	18.34	18.25
4 sl	RTP loss (%)	6.76	4.17
4 sl	PSNR diff (dB)	1.32	0.81

8 sl	TOTAL loss (%)	16.65	16.13
8 sl	RTP loss (%)	2.56	0.93
8 sl	PSNR diff (dB)	0.71	0.23

13 sl	TOTAL loss (%)	15.06	14.72
13 sl	RTP loss (%)	0.46	0.21
13 sl	PSNR diff (dB)	0.15	0.06
